# What is the degree of social disability risk in China under the background of the aging population? Social disability risk measurement index system design and evaluation research based on China

**DOI:** 10.3389/fpubh.2023.1087276

**Published:** 2023-03-13

**Authors:** Qianqian Guo, Yufeng Sun, Miao Fan, Zhichun Li

**Affiliations:** ^1^Department of Social Security, School of Public Health and Management, Ningxia Medical University, Yinchuan, China; ^2^Department of Social Medicine and Health Management, School of Public Health and Management, Ningxia Medical University, Yinchuan, China; ^3^Key Laboratory of Environmental Factors and Chronic Disease Control, Ningxia Medical University, Yinchuan, China

**Keywords:** demographic aging, risk of social disability, evaluation system, analytic hierarchy process, entropy method, China

## Abstract

**Objective:**

The impact of the aging population in China varies between regions. It is because regions with different resource endowments, such as those related to economy, population, and medical care, have different degrees of disability risk in the face of the increases in the disabled and semi-disabled older population caused by the overall aging of the population. This study aimed to construct an evaluation system to monitor and measure the degree of social disability risk in different regions in China and to evaluate and compare the degree of social disability risk in different regions using empirical data.

**Method:**

This study used the Delphi method to construct a social disability risk measurement index system with macro, meso, and micro dimensions. At the same time, based on the data of CHARLS2018, an AHP-entropy method was used to calculate the index's total weight, and the standard deviation classification method was used to classify the total and criterion-level measurement scores of 28 provinces.

**Results:**

The regional degree of social disability risk was analyzed in subdimensions. Our research indicates that China's social disability risk situation is not promising, with a general medium to high-risk level. The score of degree of social disability risk among provinces is consistent with the regional economic development level to a large extent. The risk of social disability varies significantly among the eastern and central, and western regions of China and the provinces within the three regions.

**Discussion:**

Currently, the situation facing the degree of social disability risk in China is that the overall risk level of the country is higher, and the difference between regions is significant. It is necessary to take measures to meet better the needs of the aging population and the disabled and semi-disabled older populations in a large-range, large-scale, multilevel way.

## 1. Introduction

The universal trend of increased human longevity is leading to an aging population. Due to the decline in bodily function accompanying aging alongside the appearance of functional disorders, an aging society will inevitably show a significant increase in the number of disabled and semi-disabled older people. The risk of disability and semi-disability of older people will become increasingly prominent as the aging process continues.

In 2006, the World Health Organization (WHO) believed that a disabling disease afflicted only around 500 million people worldwide (1). Six years later, however, the WHO gave the World Disability Report, which soared to one billion−15% of the world's population (2). That is twice the 2006 value. The latest WHO figure is that 1.3 billion people worldwide today−16% of the global population—have a severe disability, and the number is growing as people live longer (3). In East Asia, according to statistics published by Japan's Ministry of Health, Labor, and Welfare, the number of disabled people over 18 in Japan in 1996 was 2.933 million (4). 2001 saw 3.245 million disabled people (5), an increase of 10.6% over 1996, and in 2006 the figure rose to 3.483 million (6). In South Korea, also an East Asian country, 2.4% of the country's population was registered with a disability in 2001, gradually increasing to 4.9% in 2016 (7). Since 2013, over 40% of registered persons with disabilities in Korea are aged 65 or over (8). As one of the fastest-aging countries (9) with the largest older population (10), the double burden of an aging population and an increased chronic disease has led to a continuous increase in China's disability burden (11). Data from two national sample surveys of people with disabilities show that China's disabled population was 51.64 million in 1987 (12) and increased to 82.96 million in 2006 (13), an increase of 60.65% in 20 years and accounting for 6.34% of the country's total population. Scholars predict there will be between 108.67 million and 108.79 million disabled people in China in 2020 (14). By 2030, this number is expected to grow to 136.24 million and 136.74 million (14). In 2020, China's disabled, the older population reached 43.75 million (15). They were expected to reach 614.44 million by 2030 (15), accounting for over 57% of the total disabled population (14), with the total number of disabled older people rapidly increasing to 91.4 million in 2050 (15).

In response to the continuing increase in the number of people with disabilities due to the aging of the population and the rising prevalence of non-communicable diseases worldwide (16), the United Nations and other international organizations have taken many actions. To ensure that persons with disabilities are afforded the same rights and opportunities as others (17), the UN adopted the Convention on the Rights of Persons with Disabilities in 2006 to make visible the ‘invisible' community of persons with disabilities in the traditional UN human rights protection arena. To promote the health and wellbeing of persons with disabilities (18) and the implementation of the Convention on the Rights of Persons with Disabilities (19, 20), the World Health Organization and the World Bank jointly published the World Report on Disability in 2011, which provides a comprehensive analysis of disability issues and recommendations for national and international action. The UN's 2030 Agenda for Sustainable Development commits to “leaving no one behind” (21) and recognizes disability as a cross-cutting issue that must be considered in implementing all its goals (22).

In this context, disability has become a new social risk (23). In pre-industrial or agricultural societies, where the population's life expectancy was low, disability did not form an overall social risk. As we enter an aging population, the probability of disability rises due to the decline in older people's physical and psychological functions as they age. When older people become disabled, the demand for medical and senior care services generally increases.

In contrast, the established supply system of medical and healthcare services is not yet able to meet the needs of disabled older people. There is a severe imbalance between supply and demand, especially in developing and less developed countries. As a result, in a society with a high degree of aging, where disability leads to economic poverty for the families of disabled older people, an undersupply of health care services, and an inability of the health insurance fund to cover its expenses and other difficulties, then disability becomes a new social risk for the country or region. Internationally, in societies characterized by aging populations, it has been determined that when the proportion of the total population over 60 years old reaches 10% and the proportion of the total population over 65 years old reaches 7%, a country or region is considered an aging society. Furthermore, the proportion of the total population over 60 is taken to measure the degree of aging. A proportion of 10% is called a mildly aging society, 20% is a moderately aging society, 30% is a severely aging society, and more than 35% is a deeply aging society. However, the risk of social disability induced by aging and hyper-aging has, to date, not been explored by Chinese and international academics. Existing research has looked only at the assessment of the disability level of individuals with disabilities (24–30) or, based on the natural population growth rate, average life expectancy, and other factors, used measurement methods and the existing number of the disabled older population to predict the number of disabled older people at a certain point of time in the future (14, 15, 31–33). To date, there has been no research on the degree of risk of social disability at a national or regional level among Chinese and international academics.

In recent years, significant changes have occurred in China's population structure. The aging and hyper-aging population trend are increasing, and the rate is constantly accelerating, resulting in a severe structural imbalance (34). In order to effectively deal with the risk and challenges associated with the aging population, China established and implemented the “respond positively to population aging” strategy. In November 2021, the State Council issued Opinions on Strengthening the Work of Aging in the New Era, which put forward the strategic concept of “aging risk echelon response.” The reason for this is that an aging population, as a long-term development process, creates unbalanced risks for economic and social development, which is reflected in the fact that the impact of an aging population is not the same across all regions, areas and links, but presents characteristics of varying severity (35). This means that regions with different levels of economic, population, and medical care resources, among other factors, have different degrees of disability risk in the face of increases in the disabled and semi-disabled older population caused by an aging population. Therefore, it is necessary to construct an evaluation system to monitor and measure the degree of social disability risk in different regions and to evaluate and compare the degree of social disability risk in different regions using empirical data.

This study borrowed the Delphi method to determine an index system based on national conditions with Chinese characteristics to measure national and regional older population disability risk, and, based on a combination of the analytic hierarchy process (AHP) and entropy method (EM) empowerment method, calculated various combinations weights in the index system to synthesize the empirical data to analyze further the level of social disability risk in different areas of China.

## 2. Materials and methods

### 2.1. The measurement index system of social disability risk

The level of social disability risk depends on many factors. Any one factor is only a part of this risk and cannot reflect the whole of the risk. Given the lack of Chinese and international academic research on the degree of social disability risk in different countries or regions, no Chinese or international literature can be directly referenced in this study. Based on this, when determining the construction of the index system and formulating the index framework, this study drew on macro-, meso- and micro-level analysis perspectives in economic research to fill and enrich specific indicators of three dimensions: macro-system level, mesosystem level, and micro-system level. The principle of indicator determination was as follows: macro indicators strive to reflect the degree of aging of the population in a country or region and the total quantitative support and assistance available for the older disabled population. The meso index tries to reflect the supply and demand of medical resources for the older disabled population in a meso society. The micro index mainly reflects the degree of disability of older disabled people at the micro-individual level, using the degree of individual disability in a probability sample within a region to represent the degree of disability of the group within the area. Based on the above ideas, when initially formulated the measuring and evaluation indicators of the degree of social disability risk, the primary considerations in this study were as follows: we strove to reflect social security (36, 37), economic development level, aging population (38, 39), medical resource allocation (40), medical service provision (40, 41), education level, economic status, health status (42–44), health care expenditure, psychological status (45) and social adaptation capacity (46) and other aspects indicators suitable for Chinese national conditions; interviews were conducted with relevant personnel of government departments and experts in the field of social security and social medicine to summarize the evaluation indicators for monitoring the degree of risk of disability in China; the criteria and indicator variables for assessing the degree of individual disability were extracted from existing policy documents, especially the Long-term Care Disability Grade Assessment Criteria (Trial) jointly issued by the National Medical Insurance Administration and the Ministry of Civil Affairs in 2021. Since there has been no similar research in China or internationally, this research is exploratory. Therefore, the indicators were intended to be scientific, refined, and operable, and the data were made available without omission.

#### 2.1.1. The macro-system level

The macro level is mainly measured at the national or regional level. It is the main index used to measure the degree of social disability risk in a country or region. It includes three criteria: social security, economic development level, and degree of population aging.

1. Criteria for social security. Social security is an essential index in measuring the risk of older people's disability in a region or country. The establishment of the modern industrial society, the collapse of the natural economy, and the transformation of the commodity economy into the market economy, the socialization of production and the marketization of the economy led to land security and family security in the traditional agricultural society could no longer provide a survival foundation. Thus, laborers could no longer rely on family and land to guarantee their livelihoods, and these individual risks of workers were concentrated and transferred to society, becoming social risks. As the highest authority in the management of society, the state or government is duty-bound to assume the primary responsibility of social security and alone can implement living security for the whole society through the redistribution of national income. As an economical category, finance is a kind of economic activity with the state as the main body, used to safeguard and improve people's livelihoods, promote economic and social development, protect state security, etc. It is associated with a centralized national revenue, and the expenditure activity for part of this national income is used to meet the needs of the public in order to achieve the optimized allocation of resources, fair distribution, and economic stability and development goals. The quantitative support and assistance of the country or region provided to older disabled persons reflect the function of social management and the degree of risk of the disabled older people in the country/region. Generally, regions that spend more on social security for older disabled people have a greater social disability risk than regions that spend less on social security for older disabled people. This study selected three indicators to measure this risk: the per capita social health expenditure on disabled persons, the per capita government health expenditure on disabled persons, and the proportion of social security in the regional general public budget expenditure.
2. Criteria for the level of economic development. The regional economic development level reflects the region's degree and stage of social development. It is generally believed that a region with a high economic level, early development, and a higher degree of social development will have a higher degree of social disability. As the most effective tool and crucial index used to grasp and compare the economic development levels of different countries or regions, per capita regional GDP is often used to compare the macroeconomic operational status of different regions. Therefore, this study selected per capita regional GDP to measure regional economic development.
3. Criteria for population aging. Aging and the degree of aging continuing to increase are the initial signs of a disabled society. When longevity is shared in an aging population, the length and cycle of human life are increasingly extended, the proportion of the old-age period in the life cycle increases, the number of older people and very old people gradually increases, and the top of the “pyramid” of the population age structure constantly expands. Meanwhile, combined with the inevitable decline in bodily function as people enter old age, the continuous increase of aging leads to an increased population of disabled and semi-disabled older people, a large-scale and multilevel increase in disabled and semi-disabled older people, and an increased risk of social disability. The basic situation of an aging population can be measured *via* the population's average life expectancy, the proportion of older people in the total population, the dependency ratio of the older population, and the proportion of older disabled people in the total older population. The higher the degree of aging, the higher the social disability risk.

#### 2.1.2. The mesosystem level

The meso level is also an essential aspect of the degree of social disability risk in a region. The allocation of medical social resources to potential older disabled people and the demand for medical services for older disabled people also reflect the degree of risk of social disability in a country or region.

1. Criteria for medical resource allocation. Health resource allocation refers to the distribution and transfer of health resources collected by a country or region in different health industries or departments (47), mainly including health institutions, human resources, material resources, and other elements. As relatively scarce resources, the distribution, quantity, and structure of medical resources for older people with disabilities also reflect the basic situation of regional health services for older people, which directly relates to the health rights and interests of older people with disabilities and the degree of social disability risk. Based on the China Health Statistical Yearbook of medical and health institutions, health facilities, and the classification of health personnel, this study selected three indicators to measure the allocation of medical resources for older disabled people in different regions: the number of nursing homes and sanitariums per million older population, number of beds in nursing homes and nursing stations per million older population and number of health technicians per 1,000 older population.
2. Criteria for the provision of medical services. Based on health needs and health status, the number of health services available to disabled older people reflects their health status. The number of health services received by disabled older people in a given region reflects the degree of disability risk of the older population in that region to a certain extent. According to the availability of data and the setting of the questions on medical use in the CHARLS2018 household questionnaire, the number of outpatient visits per capita older population in the past month and the number of hospital admissions per capita older population in the past year were selected as indicators to measure the supply of medical services.

#### 2.1.3. The micro-system level

The micro level, which uses the older people's disability degree in a probability sample in the area of older population groups to represent the entire area of the degree of disability, is central to measuring the degree of risk in the regional social disability index system, comprising six aspects: level of education, economic status, health conditions, health care spending, psychological status, and social adaptability.

1. Education criterion. Many epidemiological studies have shown that education level negatively correlates with disability risk (48); people with a higher education level are significantly less likely to have a disability. Education is represented by the highest level of education the respondent has received.
2. Financial situation criterion. Economic status is an index used to measure respondents' financial income and living consumption expenditure, mainly measured by per capita disposable income, which is the primary individual factor affecting the degree of disability in older people. It is generally assumed that older people with higher income levels and better economic conditions are less likely to be disabled.
3. Health status criterion. What is a disability? The typical academic definition is the partial or complete loss of some bodily functions due to old age, disease, injury, and so on so that the ability to engage in regular activity is limited or missing (49). From this definition, it is not difficult to see that regardless of what causes a disability, its direct representation must be partial or complete bodily function damage and health problems. The fact that physical function inevitably declines in old age measures health status as a vital measurement criterion, including the activities of daily living, cognitive ability, and the number of chronic diseases. According to the relevant standards in the Long-term Care Disability Grade Assessment Criteria (Trial) jointly issued by the National Medical Insurance Administration and the Ministry of Civil Affairs in 2021, poor ability to engage in daily activities, low cognitive ability, and more types of chronic disease in the older people surveyed generally indicated a high disability grade and a profound degree of disability.
4. Criterion for healthcare expenditure. Population studies have empirically shown that medical expenditure is directly related to disability status among older people. Older people in different states of disability have different amounts of medical care expenditure, and the more severe the disability status, the greater the medical expenditure (50). Therefore, this study selected per capita healthcare expenditure to measure this criterion.
5. Psychological status criterion. With the intensification of China's aging, mental problems among older people have become increasingly prominent and the focus of academic discussion. Depression has become a common mental illness among the older population, affecting the incidence of chronic diseases and the ability to engage in daily activities and further affecting the degree and grade of disability. In this study, psychological depression was selected to measure the psychological status of the older population.
6. Social adaptability criterion. The ability to adapt socially is not only a dynamic reflection of people's responses in the face of external environmental changes. However, it is also a kind of health capital of respondents, reflecting a better physical and psychological state. It can be subdivided into three aspects: social communication, social support, learning, and adaptability.

According to the selection and description of the above indicators, based on the initial index system, following the Delphi expert consultation method and after two rounds of expert consultation, this study constructed a social disability risk measurement index system which was divided into three levels of indicators: three system-level indicators, 11 criteria-level indicators, and 21 indication-level indicators, as shown in Table 1.

**Table 1:** Measurement index system of social disability risk.

**Target layer**	**System layer**	**Criteria layer**	**Index layer**	**Unit**
Comprehensive measurement index of social disability risk	Macro-system (A1)	Social security (B1)	Per capita social health expenditure of disabled persons (C1)	Yuan
			Per capita government health expenditure of disabled persons (C2)	Yuan
			Proportion of social security in older people regional general public budget expenditure (C3)	%
		Economic development (B2)	Per capita regional GDP (C4)	Yuan
		Aging of the population (B3)	Average life expectancy of the population (C5)	Year
			Proportion of the older people in the total population (C6)	%
			Dependency ratio of the older population (C7)	%
			Proportion of the older disabled population in the older population (C8)	%
	Mesosystem (A2)	Allocation of medical resources (B4)	Number of nursing homes and sanitariums per million older population (C9)	So
			nuMber of beds in nursing homes and nursing stations per million older population (C10)	Bed
			number of health technicians per 1,000 older population (C11)	Person
		Provision of medical services (B5)	number of outpatient visits per capita older population in the past month (C12)	Times
		number of hospital admissions per capita older population in the past year (C13)	Times
	Micro-system (A3)	Education (B6)	Education level of the older people per capita (C14)	–
		Financial situation (B7)	Per capita disposable income (C15)	Yuan/person
		Health status (B8)	Activities of daily living per capita older population (C16)	–
			Cognitive ability of per capita older population (C17)	–
			Number of chronic diseases per capita in the older population (C18)	Types
		Healthcare expenditure (B9)	Per capita healthcare expenditure (C19)	Yuan
		Psychological status (B10)	Psychological status of the per capita older population (C20)	–
		Social adaptability (B11)	Social adaptability of the per capita older population (C21)	–

The determination of the measurement index system was based on the initial indicator system, strictly following the process and paradigm of the Delphi expert consultation method, after two rounds of written consultation with 15 experts, screening of the initially proposed indicators according to the boundary value method, and making additions, deletions, and corrections according to the opinions expressed in the experts' written consultations. The expert authority coefficient and Kendall coordination coefficient of the two rounds of correspondence consultation were 0.72 and 0.174, and 0.74, and 0.137, respectively, with *P*-values < 0.001 and 0.004, which passed the consistency test.

The Delphi research design method follows the “Guidelines for Conducting and Reporting Delphi Studies” (51). Moreover, the Delphi panel members were carefully selected for their diversity and “richness of information” (52).

In this study, 15 social security, health management, health policy, and social medicine experts were invited to collect information using a Delphi expert consultation form supplemented by unstructured interviews. Fifteen experts, all of whom had a master's degree or higher, including 10 with a Ph.D. (66.67%), 10 with a senior title (66.7%), and nine with more than 10 years of experience (60%; Table 2).

**Table 2:** Demographic characteristics of the Delphi panel (*N* = 15).

**Characteristics**	***n***	**Percentage (%)**
**Gender**		
Male	2	13.3
Female	13	86.7
**Age (years)**		
≤ 30	1	6.7
31–40	11	73.3
41–50	2	13.3
51–60	1	6.7
≥61	0	0
**Experience in profession (years)**		
≤ 5	1	6.7
6–10	5	33.3
11–15	7	46.7
16–20	1	6.7
21–25	0	0
≥26	1	6.7
**Professional title**		
Professor	4	26.7
Associate professor	6	40.0
Lecturer	5	33.3
**Education degree**		
Master degree	5	33.3
Doctorate	10	66.7
**Specialty**		
Social medicine	6	40.0
Health management	1	6.7
Public administration	1	6.7
Social security	2	13.3
Public service management	5	33.3
**Research direction**		
Health policy and management	1	6.7
Health economics and health policy	3	20.0
Health policy and health management	4	26.7
Medical security	1	6.7
Health services management	1	6.7
Social security	3	20.0
Health management	2	13.3

### 2.2. Data sources and index assignment

The data used in this study to measure the risk of regional social disability were taken from the 2018 China Health and Retirement Longitudinal Study (CHARLS2018), the 2019 China Health Statistical Yearbook, the 2019 China Statistical Yearbook, the Seventh National Census and the 2018 National Sample Survey on Population Changes, which was partially used to ensure data for the current year of 2018.

CHARLS, a large-scale interdisciplinary survey project hosted by the National Institute of Development at Peking University, is a tracking and longitudinal survey aimed at collecting a set of high-quality microdata representing households and individuals aged 45 and above in mainland China. To ensure international comparability and best practice, CHARLS draws on international experience in the design and measurement of specific questions, aligning with similar international studies such as the US Health and Retirement Study (HRS), the English Longitudinal Study of Aging (ELSA) and the Survey of Health, Aging, and Retirement in Europe (SHARE). To ensure a representative sample, the CHARLS National Baseline Study carried out in 2011 covered 28 provinces (municipalities and autonomous regions) and 150 county-level units, involving 450 village-level units (villages and urban communities) and covering more than 17,000 respondents from more than 10,000 households.

Since this study investigated the 28 provinces (municipalities and autonomous regions) included in the CHARLS database and used probability sampling data in the region covered by CHARLS to represent the situation of groups in the region, the 21 indicators at the index level were used as the units for the research samples. The 21 indicators were not all included in the screening conditions at one time for sample screening. The reasons are as follows: (1) The 21 index-layer indicators determined by the Delphi expert consultation method came from macro-, meso- and micro-system layers, and CHARLS cannot provide all empirical data. (2) The degree of social disability risk in 28 provinces (municipalities, autonomous regions) was analyzed and compared, with provinces as the comparison unit. There was no need to screen samples in a unified way and then classify them. (3) CHARLS's research is authoritative within China concerning older people's health data; the data quality is high, and the loss of samples is minimal, but there is still a loss of data in the database and bumping, especially for older respondents. The questionnaire involves 21 indicator problems without a complete answer, especially in the age of the sample of this study, which was higher, being 65 years old and above. If the 21 indicators were uniformly covered as the screening criteria, too many samples would have been excluded, resulting in the problem of insufficient representation. The empirical data of indicators C8, C12, C13, C14, C16, C17, C18, C20, and C21 in the third-level indicator layer were from CHARLS2018, and the specific values are shown in Table 3.

**Table 3:** Partial measure index of social disability risk and its assignment.

**Index layer**	**Index assignment**	**Unit**
Proportion of the older disabled population in the older population (C8)	Physical Self-Maintenance Scale (PSMS) of the CHARLS household questionnaire was used to determine whether the older respondent was disabled, which corresponded to the six questions DB010-DBA015 in the questionnaire. Four options: “No difficulty” or “have difficulty but can still do,” were considered “non-disability,” and “have difficulty and need help” or “cannot do” were considered “disability.” If one of the six questions was scored “disability,” the respondent was considered disabled	%
Education level of the older people per capita (C14)	The degree of education was measured by BD001_W2_4 in the CHARLS questionnaire and adopted a reverse assignment, with a total of 11 discrete variables: “Doctoral degree” = 1, “Master's degree” = 2, …, “not finish primary school” = 10, “no formal education” = 11	–
Activities of daily living per capita older population (C16)	The ADL scale in CHARLS was used to measure the activities of daily living (ADL), including six items of a physical self-maintenance scale (PSMS) and six items related to instrumental activities of daily living corresponded to DB010-DB015, DB016-DB020, and DB035 in the questionnaire. Among the options were “no difficulty” = 1, “difficulty but can still do” = 2, “difficulty and need help” = 3, “cannot do” = 4, and the older respondents who answered “no telephone” in question DB035 were excluded	–
Cognitive ability of per capita older population (C17)	Cognitive ability is measured by the simple mental state in CHARLS and consists of three parts: DC001_W4-DC012_W4, DC014_W4_DC014_W4_5, and DC016_W4-DC024_W4. There are 24 questions in total. Each correct answer is marked with 1 point, and the score ranges from 0 to 24 points	–
Number of chronic diseases per capita in the older population (C18)	The self-reported diagnosis of chronic diseases by doctors of the respondents was used as a measurement. Among the 14 chronic diseases listed in the questionnaire, one respondent was diagnosed with one, and the score ranged from 0 to 14, corresponding to the 14 questions under DA007 in CHARLS	-
Psychological status of the per capita older population (C20)	Psychological status was measured by CESD in the CHARLS questionnaire, corresponding to 10 questions DC009-DC018. Among the options were “rarely or none of the time” = 0, “some or a little of the time” = 1, “occasionally or a moderate amount of the time” = 2, and “most or all of the time” = 3, with the score ranging from 0 to 30	–
Social adaptability of the per capita older population (C21)	One point is scored for each of the 11 social interaction activities, measured by the DA056 question in CHARLS. The score ranges from 0 to 11	–

The China Health Statistical Yearbook is an annual publication reflecting the development of health undertakings and the health status of residents in China. It is also a national authoritative data source that can be used to study the development of health undertakings and the health level of residents in China. The empirical data of C1, C2, C9, C10, and C11 indicators in this study were obtained from or collated by the 2019 China Health Statistical Yearbook.

The 2019 China Statistical Yearbook systematically collected the economic and social statistics of all provinces, autonomous regions, and municipalities in 2018. It comprehensively reflects China's economic and social development during that period. The empirical data of the four indicators of C3, C4, C15, and C19 in this study were obtained from the 2019 China Statistical Yearbook.

Designed to accurately depict changes in the new era of national population development, strengthen population development strategy research, and provide a basis for promoting this research to realize long-term and balanced population development, the 2018 National Sample Survey on Population Changes was an annual nationwide sample survey organized by the National Bureau of Statistics (NBS) on November 1, 2018, with a sampling ratio of 0.820‰. The empirical data for the “Proportion of the older people in the total population (C6)” and “dependency ratio of the older population (C7)” used in this study were compiled from the sample data of the 2018 National Sample Survey on Population Changes.

It is worth noting that the “average life expectancy of the population (C5)” index could only use the national census data to ensure that the statistical methods, statistical caliber, and statistical time of the data of the 28 provinces were consistent. The national census is conducted every 10 years, wherein years ending with 0 are census years. In order to ensure the credibility of the data, the average life expectancy of each province used in this study was taken from the Seventh Census conducted in 2020.

### 2.3. A comprehensive evaluation of AHP combined with the entropy method

**Figure 1:**
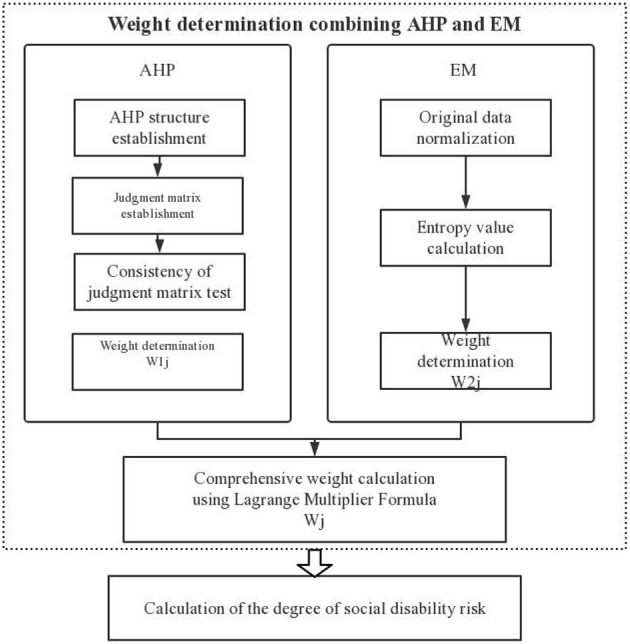
Flowchart of comprehensive evaluation combining the entropy method (EM) and analytic hierarchy process (AHP).

After synthesizing the existing relevant measurement research data from China and internationally and consulting relevant experts, this study adopted a comprehensive weighting method combining subjective and objective weighting to evaluate the index system comprehensively and combined the index weights calculated using the analytic hierarchy process (AHP) and entropy method (EM) with a Lagrange multiplier formula. The final combined weight of the regional social disability risk index was obtained to comprehensively analyze the respective degrees of social disability risk of 28 provinces (municipalities and autonomous regions) covered by the CHARLS2018 database. A flowchart of the comprehensive evaluation combining AHP and EM is presented in Figure 1.

#### 2.3.1. Weight determination using AHP

The analytic hierarchy process (AHP) is one solution to the problem of the complex multi-objective combination of qualitative and quantitative methods to calculate weights in decision-making research. Professor T. L. Saaty, an American operations research scientist, developed it. The method combines quantitative analysis with qualitative analysis, using the experience of experts and scholars to judge whether it is possible to determine the relative importance of various measured items. In addition, the weight of each index under each decision-making scheme is reasonably given, and the order of advantages and disadvantages of each scheme is calculated using that weight so that it can be more effectively applied to those topics that are difficult to address simply by using quantitative methods (53). Because the application of AHP requires less objective data, a complex monitoring and evaluation index system can still be given a better weight through subjective evaluation when the evaluation index data are difficult to collect or cannot be ultimately collected. Therefore, this method is often used to evaluate index systems in social sciences.

As part of applying the Delphi method to construct the measurement index system, 15 experts in related fields were brought together to score the importance of 21 indicators in the third-level index layer, and the value of each score itself included the relative importance of pairwise indicators in each level. Thus, this study directly used measuring software to rate the importance of 21 indicators in the index layer to calculate the average values and used the average value information to obtain the relative importance and establish the judgment matrix required by AHP (see Table 4). Thus, we calculated 21 tertiary index weights using AHP, and the results can be seen in Table 5. After consistency testing, the maximum characteristic root λmax = 21, the RI value was 1.636, and the CR value of the consistency judgment index was < 0.001. Therefore, the weighting results of the index layer analytic hierarchy process of the social disability risk measurement index system passed the consistency test.

**Table 4:** AHP judgment matrix.

**Average value**	**Item**	**C1**	**C2**	**C3**	**C4**	**C5**	**C6**	**C7**	**C8**	**C9**	**C10**	**C11**	**C12**	**C13**	**C14**	**C15**	**C16**	**C17**	**C18**	**C19**	**C20**	**C21**
8.533	C1	1	0.928	0.985	1	1.049	1	1.016	1.032	1	1	0.985	1.143	1.306	1.049	0.955	0.914	0.928	0.941	0.941	1	1.123
9.2	C2	1.078	1	1.062	1.078	1.131	1.078	1.095	1.113	1.078	1.078	1.062	1.232	1.408	1.131	1.03	0.986	1	1.015	1.015	1.078	1.211
8.667	C3	1.016	0.942	1	1.016	1.066	1.016	1.032	1.048	1.016	1.016	1	1.161	1.327	1.066	0.97	0.929	0.942	0.956	0.956	1.016	1.14
8.533	C4	1	0.928	0.985	1	1.049	1	1.016	1.032	1	1	0.985	1.143	1.306	1.049	0.955	0.914	0.928	0.941	0.941	1	1.123
8.133	C5	0.953	0.884	0.938	0.953	1	0.953	0.968	0.984	0.953	0.953	0.938	1.089	1.245	1	0.91	0.871	0.884	0.897	0.897	0.953	1.07
8.533	C6	1	0.928	0.985	1	1.049	1	1.016	1.032	1	1	0.985	1.143	1.306	1.049	0.955	0.914	0.928	0.941	0.941	1	1.123
8.4	C7	0.984	0.913	0.969	0.984	1.033	0.984	1	1.016	0.984	0.984	0.969	1.125	1.286	1.033	0.94	0.9	0.913	0.926	0.926	0.984	1.105
8.267	C8	0.969	0.899	0.954	0.969	1.016	0.969	0.984	1	0.969	0.969	0.954	1.107	1.265	1.016	0.925	0.886	0.899	0.912	0.912	0.969	1.088
8.533	C9	1	0.928	0.985	1	1.049	1	1.016	1.032	1	1	0.985	1.143	1.306	1.049	0.955	0.914	0.928	0.941	0.941	1	1.123
8.533	C10	1	0.928	0.985	1	1.049	1	1.016	1.032	1	1	0.985	1.143	1.306	1.049	0.955	0.914	0.928	0.941	0.941	1	1.123
8.667	C11	1.016	0.942	1	1.016	1.066	1.016	1.032	1.048	1.016	1.016	1	1.161	1.327	1.066	0.97	0.929	0.942	0.956	0.956	1.016	1.14
7.467	C12	0.875	0.812	0.862	0.875	0.918	0.875	0.889	0.903	0.875	0.875	0.862	1	1.143	0.918	0.836	0.8	0.812	0.824	0.824	0.875	0.982
6.533	C13	0.766	0.71	0.754	0.766	0.803	0.766	0.778	0.79	0.766	0.766	0.754	0.875	1	0.803	0.731	0.7	0.71	0.721	0.721	0.766	0.86
8.133	C14	0.953	0.884	0.938	0.953	1	0.953	0.968	0.984	0.953	0.953	0.938	1.089	1.245	1	0.91	0.871	0.884	0.897	0.897	0.953	1.07
8.933	C15	1.047	0.971	1.031	1.047	1.098	1.047	1.063	1.081	1.047	1.047	1.031	1.196	1.367	1.098	1	0.957	0.971	0.985	0.985	1.047	1.175
9.333	C16	1.094	1.014	1.077	1.094	1.148	1.094	1.111	1.129	1.094	1.094	1.077	1.25	1.429	1.148	1.045	1	1.014	1.029	1.029	1.094	1.228
9.2	C17	1.078	1	1.062	1.078	1.131	1.078	1.095	1.113	1.078	1.078	1.062	1.232	1.408	1.131	1.03	0.986	1	1.015	1.015	1.078	1.211
9.067	C18	1.063	0.986	1.046	1.063	1.115	1.063	1.079	1.097	1.063	1.063	1.046	1.214	1.388	1.115	1.015	0.971	0.986	1	1	1.063	1.193
9.067	C19	1.063	0.986	1.046	1.063	1.115	1.063	1.079	1.097	1.063	1.063	1.046	1.214	1.388	1.115	1.015	0.971	0.986	1	1	1.063	1.193
8.533	C20	1	0.928	0.985	1	1.049	1	1.016	1.032	1	1	0.985	1.143	1.306	1.049	0.955	0.914	0.928	0.941	0.941	1	1.123
7.6	C21	0.891	0.826	0.877	0.891	0.934	0.891	0.905	0.919	0.891	0.891	0.877	1.018	1.163	0.934	0.851	0.814	0.826	0.838	0.838	0.891	1

**Table 5:** The indicator weight of the social disability risk measurement index.

**Target layer**	**System layer**	**Weight**	**Criteria layer**	**Weight**	**Index layer**	**Attribute**	**Weight**		
							**AHP**	**EM**	**Comprehensive**
Comprehensive measure index of social disability risk	Macro-system (A1)	0.39808	Social security (B1)	0.18643	C1	Positive	0.04798	0.0991	0.07454
					C2	Positive	0.05172	0.0627	0.06155
					C3	Positive	0.04873	0.0445	0.05034
			Economic development (B2)	0.05404	C4	Neutral	0.04798	0.0521	0.05404
			Aging of the population (B3)	0.15761	C5	Positive	0.04573	0.0201	0.03277
					C6	Positive	0.04798	0.0242	0.03683
					C7	Positive	0.04723	0.0302	0.04082
					C8	Positive	0.04648	0.041	0.04719
	Mesosystem (A2)	0.30567	Allocation of medical resources (B4)	0.23029	C9	Positive	0.04798	0.0873	0.06996
					C10	Positive	0.04798	0.2201	0.11108
					C11	Positive	0.04873	0.0426	0.04925
			Provision of medical services (B5)	0.07539	C12	Positive	0.04198	0.0335	0.04054
					C13	Positive	0.03673	0.0283	0.03485
	Micro-system (A3)	0.29624	Education (B6)	0.02181	C14	Positive	0.04573	0.0089	0.02181
			Financial situation (B7)	0.06303	C15	Neutral	0.05022	0.0677	0.06303
			Health status (B8)	0.09307	C16	Positive	0.05247	0.0093	0.02388
					C17	Reverse	0.05172	0.0203	0.03502
					C18	Positive	0.05097	0.0196	0.03417
			Healthcare expenditure (B9)	0.0465	C19	Positive	0.05097	0.0363	0.0465
			Psychological status (B10)	0.04747	C20	Positive	0.04798	0.0402	0.04747
			Social adaptability (B11)	0.02437	C21	Reverse	0.04273	0.0119	0.02437

The index weight of the criterion layer is obtained by simply adding the weights of each index layer under the combination method. The index weight of the system layer is obtained by simply adding the index weights of the criterion layer.

#### 2.3.2. Weight determination using EM

AHP based on subjective evaluation can easily produce significant inaccuracies and even errors. In particular, when a subjective evaluation based on experience, intuition, and understanding significantly differs from objective facts, this judgment thinking method's calculation process and results show low accuracy. Therefore, this study also applied the entropy method, an objective weighting method with high objectivity and rationality, reducing human factors' interference. Entropy is a measure of uncertainty. The entropy method borrows the physical concept of entropy in thermodynamics, using the information carried by entropy, combined with the degree of variation of each index, and uses the tool of information entropy to calculate the objective weight of each index. Generally, the greater the amount of information, the smaller the uncertainty and entropy, the higher the degree of aggregation of the index, the more significant the role of the index in representing the entire evaluation system, and the greater the weight (54).

Due to the inconsistency of data direction and unit among indicators, before using the entropy method, it was necessary to carry out dimensionless processing on all data, that is, to adopt the normalization method: forward index forward, reverse index reverse. Since the value range of normalized data is 0~1, and a zero value cannot be used to calculate a logarithm in an entropy algorithm, non-negative translation was selected for dimensionless data. The coordinate translation formula was as follows:


(1) $$\begin{array}{l} Y_{ij}=Z_{ij}+0.0001 \end{array}$$

where *i* is the number of the province to be measured (*i* = 1, 2, ..., 28), *j* is the number of the indicator layer indicator (*j* = 1, 2, ..., 21), *Y*_*ij*_ is the value of the normalized data shifted by 0.0001 units to the right, and 0.0001 is the translation amplitude.

According to the definition of entropy, the proportion of the *j*th index in the *i*th province in the calculation of entropy value can be obtained as follows:


(2) $$\begin{array}{l} p_{ij}=\frac{Y_{ij}}{\sum_{i=1}^{28}Y_{ij}} \end{array}$$

The information entropy value of item j can be expressed as follows:


(3) $$\begin{array}{l} E_{j}=-\frac{1}{\ln\;28}\overset{28}{\underset{i=1}{\sum }}p_{ij}\cdot \ln\;p_{ij} \end{array}$$

The objective weight of the jth index can then be calculated as follows:


(4) $$\begin{array}{l} W_{2j}=\frac{1-E_{j}}{\sum_{j=1}^{21}\left(1-E_{j}\right)} \end{array}$$

#### 2.3.3. Comprehensive weight determination using the Lagrange multiplier formula

Combined weighting was carried out according to the Lagrange multiplier formula using the following Equation:


(5) $$\begin{array}{l} W_{j}=\frac{\sqrt{W_{1j}\cdot W_{2j}}}{\sum_{j=1}^{21}\sqrt{W_{1j}\cdot W_{2j}}} \end{array}$$

where *W*_1*j*_ is the emotional weight in the analytic hierarchy process, *W*_2*j*_ is the objective weight for the entropy method, and *W*_*j*_ is the combined weight after combining the main objective weights. The final weighting results are shown in Table 5.

The combined weight and dimensionless translation data were combined for calculation. Finally, the comprehensive social disability risk score of the *i*th province in 2018 could be obtained, as shown in Equation (6):


(6) $$\begin{array}{l} V_{i}=\overset{21}{\underset{j=1}{\sum }}W_{j}\cdot Y_{ij} \end{array}$$

where *i* is the number of the province to be measured (*i* = 1, 2, ..., 28).

## 3. Results

### 3.1. Contribution of each index to the degree of social disability risk

Based on the macro-level, meso-level, and micro-level analysis perspectives in economic research, a theoretical framework for measuring the social disability risk was constructed with three essential elements as the primary evaluation dimensions: the macro-national or regional level, the meso medical industry resource allocation level and the micro individual disability level of the older people. In this study, the weight coefficients of the three first-level indicators were 0.39808, 0.30567, and 0.29624, with relatively similar values, indicating that these three aspects are essential for monitoring the degree of risk of social disability. Among the second-level indicators, the weights of “social security,” “population aging,” and “medical resource allocation” were all above 0.15, which were significantly higher than other second-level items, indicating that these three items had the most extraordinary relationship with the risk of social disability. The scores of 21 three-level indicators were relatively well-proportioned, and the weight coefficients of 50% of the indicators ranged from 0.03485 to 0.05404. Among them, the two indexes with the highest coefficients were “number of beds in nursing homes and nursing stations per million older population (C10)” (0.11108) and “per capita social health expenditure of disabled persons (C1)” (0.07454).

### 3.2. Empirical analysis of measurement of social disability risk in China

By applying the above research methods, comprehensive measurement scores of the degree of social disability risk in 28 provinces (municipalities and autonomous regions) in China in 2018 could be obtained, as shown in Table 6. The higher the total score, the higher the degree of risk of social disability in the region. To more intuitively show the spatial distribution of provinces with different degrees of social disability risk, according to the standard deviation classification method ($\bar{x}$ is the average value, *S* is the standard deviation), the 28 provinces in this study were classified and divided into four levels: low-risk area (0, $\bar{x}-s$), medium-risk area ($\bar{x}-s$, $\bar{x}$), higher-risk area ($\bar{x}$, $\bar{x}+s$) and highest-risk area ($\bar{x}+s,$1). The results are shown below.

**Table 6:** The score of comprehensive measurement of the degree of social disability risk for 28 provinces (municipalities and autonomous regions) in China in 2018.

**Province**	**C1**	**C2**	**C3**	**C4**	**C5**	**C6**	**C7**	**C8**	**C9**	**C10**	**C11**	**C12**	**C13**	**C14**	**C15**	**C16**	**C17**	**C18**	**C19**	**C20**	**C21**	**Comprehensive scores**	**Ranking**
Shanghai	0.05435	0.04196	0.00449	0.05145	0.03277	0.03587	0.03165	0.01868	0.04856	0.11109	0.01052	0.02677	0	0.00851	0.06303	0.0108	0.00274	0	0.04232	0	0.00379	0.59938	1
Beijing	0.07454	0.06156	0.00452	0.05405	0.03254	0.01886	0.01369	0.00747	0.00894	0.00255	0.04925	0.04054	0.01992	0	0.06058	0.01312	0	0.01943	0.0465	0.00153	0	0.5296	2
Jiangsu	0.02058	0.00861	0.00482	0.04162	0.02037	0.03285	0.03159	0.01221	0.06996	0.0851	0.00849	0.01541	0.00914	0.0185	0.02782	0.01293	0.01013	0.007	0.02078	0.00658	0.01907	0.48356	3
Zhejiang	0.02213	0.0098	0.00287	0.03342	0.02371	0.02683	0.02456	0	0.03845	0.03235	0.01901	0.01245	0.00586	0.0186	0.03827	0.0103	0.01297	0.00807	0.02166	0.00156	0.01449	0.37738	4
Xinjiang	0.01416	0.01843	0.00555	0.00901	0.00627	0	0	0.03774	0.00553	0.00012	0.0445	0	0.03485	0.01321	0.00542	0.02388	0.0224	0.03417	0.01212	0.04748	0.02355	0.3584	5
Liaoning	0.00888	0.00007	0.05034	0.01324	0.01791	0.03598	0.03204	0.02442	0.01284	0.00432	0.00485	0.00523	0.01356	0.01401	0.01649	0.01501	0.01144	0.01018	0.0257	0.01446	0.02082	0.3518	6
Chongqing	0.01051	0.01346	0.02094	0.01718	0.01745	0.03366	0.0356	0.01203	0.01273	0.00628	0.00501	0.01195	0.01616	0.0188	0.01202	0.0129	0.01938	0.0109	0.0135	0.02831	0.02154	0.35031	7
Tianjin	0.01877	0.0187	0.01892	0.04437	0.02797	0.01732	0.01196	0.01176	0.00329	0.00001	0.01609	0.01427	0.00048	0.01608	0.02973	0.01542	0.01745	0.01297	0.03428	0.00789	0.01181	0.34954	8
Shandong	0.01102	0.00315	0.00799	0.02231	0.01983	0.03684	0.04083	0.02609	0.0193	0.00941	0.00638	0.00678	0.00674	0.01815	0.01582	0.01617	0.01446	0.00915	0.01283	0.00647	0.02181	0.33152	9
Sichuan	0.00813	0.00835	0.02075	0.00872	0.01449	0.03604	0.03802	0.01933	0.00098	0.0014	0.00379	0.01987	0.01675	0.02008	0.00672	0.01656	0.02449	0.01131	0.01163	0.02166	0.0207	0.32975	10
Inner Mongolia	0.00734	0.01524	0.01426	0.01835	0.0136	0.0124	0.00856	0.03566	0.01734	0.00339	0.02661	0.00599	0.01772	0.01538	0.0147	0.01946	0.01147	0.01556	0.01733	0.01397	0.01658	0.32092	11
Qinghai	0.0047	0.05026	0.01249	0.00812	0.00623	0.00197	0.00076	0.00409	0.0077	0.00667	0.04323	0.00862	0.01275	0.02138	0.00442	0.01916	0.0223	0.01003	0.01722	0.03328	0.0222	0.31757	12
Hubei	0.0069	0.00574	0.01852	0.01752	0.01529	0.02456	0.02326	0.01142	0.00231	0.00186	0.01175	0.00867	0.01435	0.01777	0.01124	0.01593	0.02505	0.01227	0.01856	0.02452	0.02031	0.30782	13
Jilin	0.00689	0.00951	0.02018	0.01206	0.01687	0.024	0.02035	0.03889	0.01638	0.00253	0.01124	0.00336	0.00495	0.01394	0.00717	0.01875	0.01357	0.00872	0.02069	0.01142	0.02324	0.30471	14
Guangdong	0.01308	0.01472	0.00001	0.02734	0.02033	0.0051	0.00278	0.00231	0.01859	0.00701	0.0305	0.02718	0.01764	0.01791	0.02474	0.01233	0.01812	0.00611	0.01065	0.00588	0.01851	0.30082	15
Gansu	0.00072	0.01277	0.01071	0.00001	0.00623	0.01915	0.01872	0.04585	0.00402	0.00001	0.00958	0.01641	0.01711	0.01984	0.00001	0.02115	0.01738	0.01245	0.01174	0.02592	0.02091	0.29066	16
Hebei	0.00414	0.00386	0.01451	0.00816	0.01433	0.02545	0.02691	0.03051	0.00001	0.00126	0.00614	0.02693	0.01249	0.01506	0.00805	0.01828	0.01031	0.01523	0.01105	0.01699	0.01656	0.28625	17
Anhui	0.00185	0.00613	0.01395	0.00813	0.01514	0.02782	0.02992	0.01167	0.00966	0.00815	0	0.01039	0.01461	0.02181	0.00877	0.01747	0.02153	0.01386	0.00458	0.01892	0.02076	0.28514	18
Shanxi	0.00236	0.00539	0.0172	0.00695	0.01495	0.01458	0.01225	0.04719	0.00838	0.00209	0.0182	0.00779	0.00582	0.01712	0.00608	0.0219	0.01468	0.00878	0.01299	0.02016	0.01702	0.28187	19
Shaanxi	0.01083	0.01252	0.01521	0.01596	0.01453	0.01842	0.01568	0.01242	0.00525	0.0018	0.02711	0.01515	0.01139	0.01469	0.00681	0.01418	0.00995	0.01319	0.01532	0.00819	0.01817	0.27675	20
Heilongjiang	0.00768	0.00001	0.03475	0.00593	0.01625	0.02328	0.01839	0.03227	0.00291	0.00162	0.00758	0.00244	0.01028	0.01178	0.00708	0.01819	0.00761	0.01411	0.02526	0.01483	0.01272	0.27498	21
Hunan	0.004	0.00641	0.01429	0.01073	0.01483	0.02453	0.02669	0.00843	0.00274	0.00219	0.00812	0.01189	0.0143	0.01577	0.01047	0.01553	0.01787	0.0122	0.01443	0.01405	0.01258	0.26204	22
Henan	0.00168	0.00516	0.0127	0.00935	0.01376	0.01794	0.02009	0.02651	0.0027	0.00165	0.01399	0.01555	0.01422	0.01785	0.00605	0.01553	0.01565	0.01189	0.01107	0.01042	0.017	0.26077	23
Fujian	0.00949	0.01053	0.00029	0.02972	0.01718	0.01073	0.00872	0.0067	0.01447	0.00261	0.0197	0.0239	0.00683	0.02066	0.02046	0.01398	0.00981	0.00593	0.0048	0.00198	0.02159	0.26007	24
Yunnan	0.00235	0.01285	0.01226	0.00288	0	0.01113	0.00996	0.01873	0.00571	0.00036	0.01903	0.01891	0.01436	0.02161	0.00351	0.01629	0.02276	0.00984	0.00548	0.02343	0.02438	0.25584	25
Guizhou	0.00131	0.01746	0.00312	0.00492	0.00454	0.01926	0.02251	0.01842	0.00459	0.00193	0.01519	0.00578	0.00357	0.02121	0.00128	0.01343	0.03503	0.00801	0.00171	0.01541	0.01935	0.23801	26
Jiangxi	0.00001	0.01491	0.01085	0.008	0.01391	0.01187	0.01215	0.00532	0.00396	0.00058	0.01106	0.01514	0.01495	0.01785	0.0089	0.01393	0.01196	0.00825	0	0.01724	0.01738	0.21821	27
Guangxi	0.00176	0.01011	0.01385	0.00505	0.01552	0.01321	0.0148	0.00751	0.00612	0.00331	0.01883	0.01603	0.01135	0.01748	0.0054	0	0.01093	0.00875	0.00746	0.01667	0.01333	0.21748	28

The CHARLS survey covered only 28 provincial-level administrative regions, excluding Hainan, Tibet, Ningxia, Taiwan, Hong Kong, and Macao.

#### 3.2.1. Overall analysis

**Figure 2:**
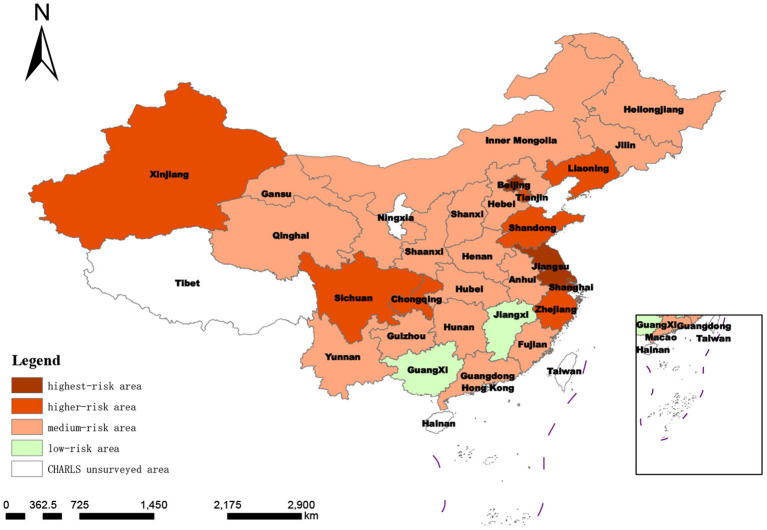
Spatial distribution of degree of regional social disability risk based on target-layer score.

It can be seen from Figure 2 that Jiangxi and Guangxi are two provincial-level regions with a low risk of social disability, accounting for 7.14% of the studied regions. There were 16 provinces (57.14%) in the medium disability risk range. There were seven provinces with high risk, accounting for 25%. Three provinces were in the highest-risk zone, Shanghai, Beijing, and Jiangsu, accounting for 10.71%. The average degree of social disability risk in 28 provinces was 0.32218, and the median degree of social disability risk was 0.302765. The proportion of provinces with a moderate or above risk was as high as 92.86%. Therefore, China's social disability risk is generally moderately high.

According to the regional division, all 12 provinces in the east covered by the survey were defined as medium or higher-risk areas, especially Shanghai, Beijing, and Jiangsu provinces, with the top three social disability risks in China. In the central region, Jiangxi was a low-risk area, and the other five provinces were all medium-risk areas. The degrees of disability risk in the western provinces spanned an extensive range, including low-, medium- and high-risk areas. It can be seen that the social disability risk scores of provinces were consistent with the regional economic development level to a large extent.

#### 3.2.2. System layer comparison and analysis

**Figure 3:**
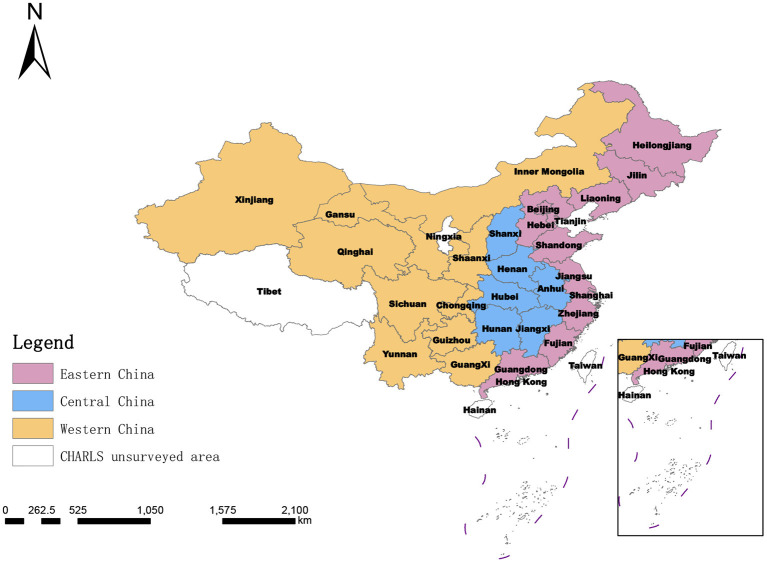
The eastern, central, and western regions of China.

**Figure 4:**
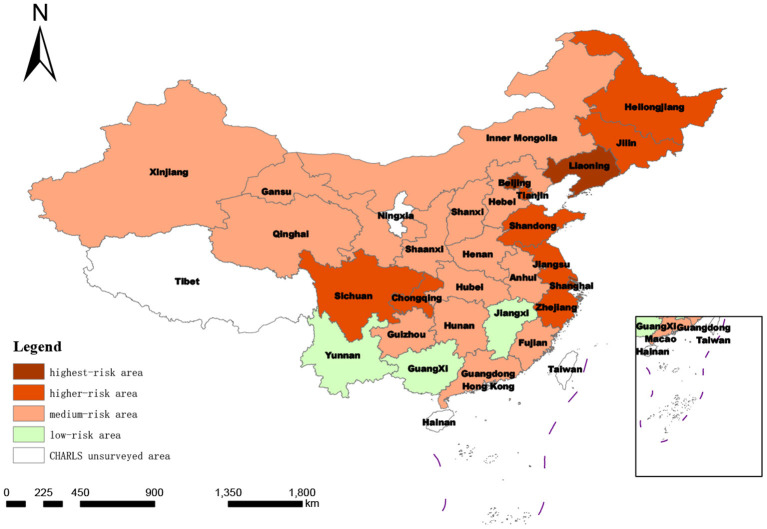
Spatial distribution of degree of regional social disability risk according to macro-system score.

**Figure 5:**
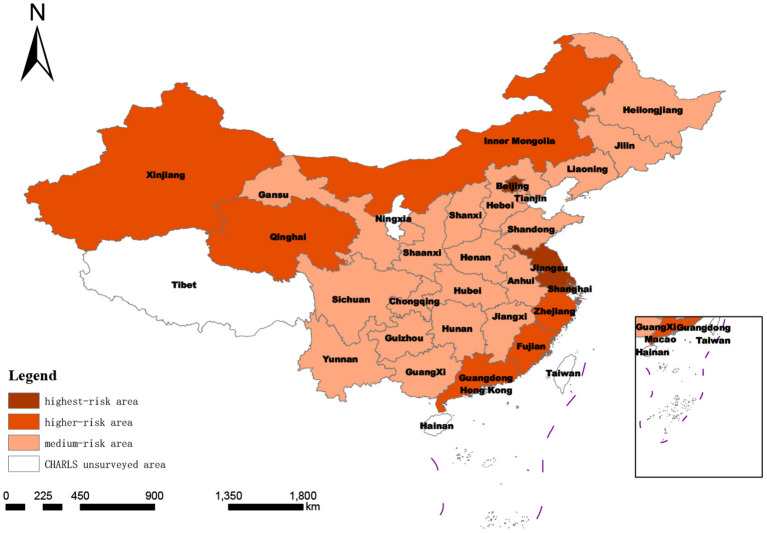
Spatial distribution of degree of regional social disability risk under mesosystem score.

**Figure 6:**
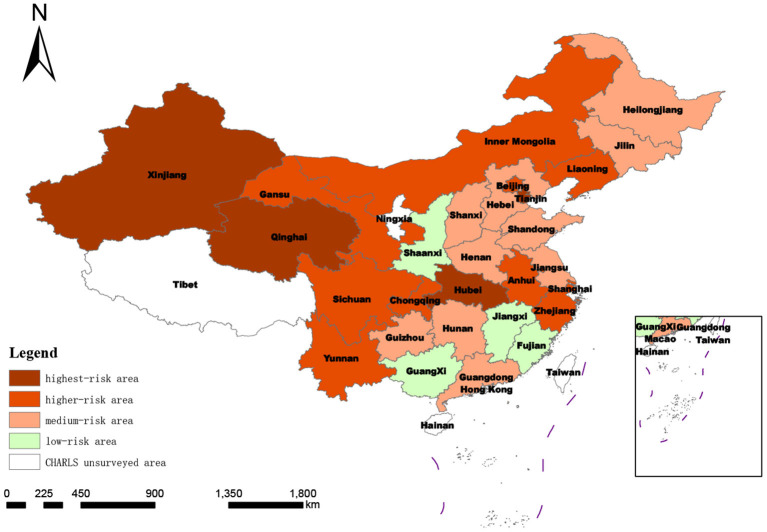
Spatial distribution of degree of regional social disability risk based on micro-system score.

The scores and distribution of the comprehensive measurement of the degree of social disability risk shown in Table 6 and Figure 3 were synthesized by summing the evaluation scores of 21 indicators at the index layer, which was too general. In order to further compare and analyze the scores of different dimensions within each province, it was necessary to conduct a more detailed analysis of the 28 provinces, as shown in Table 7 and Figures 4–6.

**Table 7:** Scores of system-layer measures of the degree of social disability risk for 28 provinces (municipalities and autonomous regions) in China in 2018.

**Province**	**Macro- system**	**Ranking**	**Meso- system**	**Ranking**	**Micro- system**	**Ranking**	**Target layer**	**Ranking**
Shanghai	0.27122	1	0.19695	1	0.13121	8	0.59938	1
Beijing	0.26723	2	0.12121	3	0.14116	5	0.5296	2
Liaoning	0.18289	3	0.0408	22	0.12812	10	0.3518	6
Jiangsu	0.17264	4	0.18811	2	0.12281	15	0.48356	3
Tianjin	0.16977	5	0.03415	26	0.14562	4	0.34954	8
Shandong	0.16804	6	0.04861	14	0.11486	19	0.33152	9
Chongqing	0.16083	7	0.05214	13	0.13735	6	0.35031	7
Sichuan	0.15382	8	0.04279	20	0.13314	7	0.32975	10
Jilin	0.14873	9	0.03846	25	0.11752	17	0.30471	14
Zhejiang	0.14332	10	0.10813	4	0.12592	13	0.37738	4
Heilongjiang	0.13857	11	0.02483	28	0.11158	22	0.27498	21
Hebei	0.12788	12	0.04683	17	0.11153	23	0.28625	17
Inner Mongolia	0.12542	13	0.07105	8	0.12445	14	0.32092	11
Hubei	0.12321	14	0.03894	24	0.14567	3	0.30782	13
Shanxi	0.12087	15	0.04228	21	0.11873	16	0.28187	19
Shaanxi	0.11556	16	0.06069	10	0.1005	25	0.27675	20
Anhui	0.11461	17	0.04281	19	0.12772	11	0.28514	18
Gansu	0.11414	18	0.04713	16	0.12939	9	0.29066	16
Hunan	0.10991	19	0.03923	23	0.1129	21	0.26204	22
Henan	0.10718	20	0.04811	15	0.10548	24	0.26077	23
Fujian	0.09335	21	0.06751	9	0.09921	26	0.26007	24
Guizhou	0.09154	22	0.03105	27	0.11542	18	0.23801	26
Xinjiang	0.09117	23	0.085	6	0.18223	1	0.3584	5
Qinghai	0.08862	24	0.07897	7	0.14998	2	0.31757	12
Guangdong	0.08567	25	0.10092	5	0.11423	20	0.30082	15
Guangxi	0.08182	26	0.05564	12	0.08002	28	0.21748	28
Jiangxi	0.07701	27	0.04569	18	0.09551	27	0.21821	27
Yunnan	0.07018	28	0.05837	11	0.12729	12	0.25584	25

##### 3.2.2.1. Macro-system measurement

As shown in Table 7, the score ranking at the macro level was similar to the comprehensive ranking: of the top 10 provinces in the macro ranking, Jilin was an exception (ranked 14th in the comprehensive ranking); the total scores of the other nine provinces were all in the top 10 and Shanghai and Beijing were ranked first and second in both the macro ranking and comprehensive ranking. The standard deviation classification results of the measurement score at the macro system level showed that compared with the comprehensive score of degree of risk classification, the number of provinces defined as highest-risk areas remains the same (Jiangsu province moved from highest risk to higher risk, while Liaoning province moved from higher risk to highest risk), the number of provinces in the higher-risk category increased from seven to eight (Jilin and Heilongjiang provinces moved to higher risk; Xinjiang from higher to medium risk). The number of medium-risk provinces was reduced by 2 to 14, and the number of low-risk provinces increased to three by including Yunnan province in the western region.

##### 3.2.2.2. Meso-system measurement

At the meso level, the risk of social disability in the 28 provinces (municipalities and autonomous regions) covered by CHARLS2018 no longer included low-risk areas after classification according to the standard deviation of this dimension. This indicates that if the risk of social disability in the region is measured only by the dimension of the demand and supply of medical resources for the older disabled population, then the national risk of disability investigated in this study will be upgraded and increased, and the focus will be on the medium-risk level. In this subdivision, the number of provinces at medium risk increased from 16 to 19. In contrast, the number of provinces defined as higher risk and highest risk areas did not change much, and the number of provinces with higher risk decreased from 7 to 6.

##### 3.2.2.3. Micro-system measurement

From the micro-system standard deviation classification features, three areas in China were included as risk areas. This indicates that the regional differences in the degree of social disability risk measured at the micro level are more evident in China than in other scales. The differences among provinces in the eastern, central, and western regions are significant.

## 4. Discussion

Looking back at the history of human societies, the development of longevity, fewer children, and urbanization has led to an aging trend in society. European scholars first began to explore the economic impact of changing age structures. Then the international community paid increasing attention to the aging phenomenon and its impact on human societies, and aging research emerged, grew, and flourished. The risk of aging under the phenomenon of population aging, which marks the arrival of an aging society, has evolved gradually as forms of aging have developed. As aging progresses, the population's life expectancy generally increases, the older population gradually grows, and the top of the population age pyramid continues to expand. At the same time, the deepening of aging presents an increase in the number of disabled and semi-disabled older people due to the physiological rule that people's physical functions inevitably decline when they enter old age. According to the results of the seventh census, there are 264 million people aged 60 and above in China, accounting for 18.7% of the total population, making it the country with the largest older population in the world. At the same time, there are currently 43.75 million disabled, older people in China. With such a severely aging population and many disabled, older people, what is the risk of social disablement in China? How is it measured? What tools are used to measure it?

This study first constructs a system of indicators to measure the risk of social disablement in China, which involves complex multi-dimensional variables such as social security, healthcare resource supply and demand, and the health status of the older population. It is an extensive system with dimensions including the country or region at the macro level, the healthcare allocation at the meso level, and the individual disabled older people at the micro level.

Secondly, after constructing the measurement index system, this study used a comprehensive weighting method combining hierarchical analysis and the entropy method to calculate the weights of each indicator. The study results show that, among the three system-level indicators, the macro system has the highest weight, followed by the mesosystem, and the microsystem has the lowest weight. It shows that the quantitative national or governmental support and assistance for social security for the older population also reflects the level of risk of old age disability in the region. The importance of public programs in improving the situation of people with disabilities is highlighted in line with previous reports (55, 56). Regions with high spending on social security for older disabled people are at greater risk of social disablement than regions with low spending on social security for older disabled people. However, the three values are relatively close, suggesting that these three areas are crucial for monitoring the level of risk of social disablement. Previous studies all support the importance of health resource utilization (57, 58) in improving the situation of people with disabilities and the geographical differences (59) in the prevalence of disability among older people.

Among the indicators in the criteria layer, the highest overall weighting coefficient is for “allocation of medical resources (B4)” at 0.23029, the lowest is for “education (B6)” at 0.02181, and the median is for “financial situation (B7)” at 0.6303. The top three coefficients are “allocation of medical resources (B4),” “social security (B1),” and “aging of the population (B3).” These, which all weigh 0.15 or more, are significantly higher than the other criteria layer indicators, indicating that these three indicators have the most significant relationship with the risk of social disablement and present a higher coefficient. Two of the top three indicators belong to the macro level, confirming our discussion above that national or regional actions significantly impact social disablement risk.

The 21 indicators scored relatively evenly in the indicator tier with a standard deviation of 0.020544. Regarding relative values, the highest weighting coefficient was C10 at 0.11108, and the lowest was C14 at 0.02181, a difference of nearly five times. This indicates that in this study, the “number of beds in nursing homes and nursing stations per million older population (C10)” has the most significant impact on the level of social disablement risk in the region. In contrast “education level of the older people per capita (C14)” has the slightest effect. Previous research (60) has suggested that the more educated older person is, the more knowledgeable they are about primary health care compared to less educated older people. When health problems arise, more educated older people are more likely to be proactive in their treatment (61), thus avoiding a significant reduction in their ability to take care of themselves. However, the results of this study show that the education level of the older population has less impact on the level of risk of social disablement. This may be because older Chinese people, whose standard of living and economic income have increased significantly due to health education, are more physically active, take care of their health seriously, regardless of their education level, and receive treatment when they are unwell.

Afterward, the combined weights of the indicators and the dimensionless processed empirical data were multiplied to derive the risk level of social disablement in 28 provincial administrative regions in China. In order to visualize the magnitude of risk in different provinces, the standard deviation grading method was used to classify the risk scores of the 28 provinces studied above into four levels: low risk, medium risk, higher risk, and high-risk zones, concerning relevant studies (62). Then, the analysis was carried out in the overall and system-level dimensions. The spatial distribution of social disablement risk in Chinese provinces under different dimensional analyses was mapped using ArcGIS version 10.2 software.

In this study, the overall analysis includes all 21 indicator layers indicators. The results show that at this time, except for Jiangxi and Guangxi, all other 26 provinces are in the medium and above risk zone for social disablement risk, accounting for 92.86%. This indicates that the overall risk of social disablement in China is at a moderate to high-risk level, and the situation is not optimistic. The reason for this may be the rising life expectancy of China's population due to improved living standards and medical technology, which has led to a continuous increase in the degree of aging and a rise in the size and proportion of potentially disabled older people. This, coupled with the lagging change in health concepts, unhealthy living and eating habits, and the lack of fitness facilities, as well as the natural pattern of physical decline in old age, has led to a rapid increase in the number of disabled and semi-disabled people in China's aging population, resulting in a high risk of disability in Chinese society. Through a more detailed analysis, it was found that Jiangxi and Guangxi provinces have a higher risk of social disability in terms of “economic development (B2),” “aging of the population (B3),” “health status (B8)” and “healthcare expenditure (B9).” We believe combining these factors has contributed to the low risk of social disablement in Jiangxi and Guangxi provinces. Lower levels of economic development and lower levels of social development also result in a smaller size and share of the older, more senior population. The higher number of younger older people means that older people are in relatively good health and have higher levels of activity in daily living and cognitive ability. In surveying the prevalence of chronic diseases in the older population, CHARLS uses respondents' self-reported doctor-diagnosed chronic diseases. With a low level of economic development, older people have less disposable income and are less willing to be treated by doctors when they are ill, so naturally, there are fewer doctor-diagnosed chronic diseases, and the health status of the older population collected from the survey is relatively good.

By region, the results show that all five provinces in the central region, except for Jiangxi, are in the medium risk zone, while all 12 eastern provinces are in the medium and higher risk zone, especially in Shanghai, Beijing, and Jiangsu, the three most economically developed provinces in China, which are among the top three in the country. This suggests that the level of social disablement risk in Chinese provinces coincides to a greater extent with the level of regional economic development. This may be because regions with higher levels of economic development are also regions with earlier development and higher levels of social development; where medical technology is advanced, life expectancy is relatively high. The older population is also more extensive, with a higher degree of population aging and naturally higher levels of social disablement risk. Previous studies (63, 64) confirm China's positive correlation between economic development and population aging.

When only the macro-system level is measured in the system level dimension analysis, the results show that the macro-system level social disablement risk scores of the 28 measured provinces in China are the most similar to the overall analysis ranking. This indicates the highest weighting of the macro-system level indicators, reflecting the combined weighting of the subjective Delphi expert consultation method and the objective entropy value method. The mesosystem indicators measure healthcare resource allocation and healthcare service provision. When only the mesosystem level is measured, social disablement risk in China will escalate and increase. There will no longer be provinces with low levels of social disablement risk. This may be because disabled older people receive more outpatient or inpatient treatment than non-disabled people, and social healthcare resources are rationed higher for the older disabled. When only the microsystem level is measured, provinces in the three regions of East, West, and Central China cover low, medium, higher, and high-risk zones. This reflects that provinces within the three regions of East, West, and Central China vary more significantly when the level of disablement of the sampled older people is used to reflect the regional level of risk of social disablement. This may be because even within the same region, there are still considerable differences in socio-economic development (65) and demographic differences (66, 67) between provinces, resulting in different levels of disability among older people in different provinces within the same region.

As seen from the above, currently, the situation facing the degree of social disability risk in China is that the overall risk level of the country is higher, and the difference between regions is significant. It is necessary to take some measures to meet better the needs of the aging population and the disabled and semi-disabled older populations in a large-range, large-scale, multilevel way.

First, it is necessary to change our ideas and reshape our cognition. As the initiating factor of disability risk in the older population, population aging is the current trend (68), and the risks and challenges it brings are global (69), long-term (70), and irreversible (71). Therefore, in the face of the challenge of social disability risk, we must abandon the unsuitable concepts of “partial war,” “quick decision war,” and “temporary war.” This is an inevitable historical turning point in the development of human society from the perspectives of both the evolution of research disciplines and our understanding of the causes of aging. Based on this, in order to realize the transformation from problem-based research to social-form research and from solving problems to constructing a new social form, research perspectives should also expand from simply focusing on the problem of disabled and semi-disabled older people to structural problems and social form problems and explore the rise and fall of an aging society.

Second, institutional guarantees are required. Establishing and improving the old-age security system, especially the multilevel old-age service system based at home, supported by the community and supplemented by institutions, will be the foundation for ensuring the quality of life among the disabled and semi-disabled older population. The establishment and improvement of old-age social security, medical security, and long-term care service systems are the three pillars of old-age security and old-age service. Legal measures should be adopted to protect every citizen's fundamental rights and interests, including the disabled and semi-disabled older people.

Third, it is necessary to adhere to the “all-round considerations and arrangements for the nation as a whole” and “give full play to the initiative of both the central and local governments.” Because of the high social disability risk across the whole country and the significant differences in risk between regions, it is necessary, on the one hand, to strengthen the top-level design at the national level—that is, all regions and departments functioning under the unified leadership of a central, overall arrangement, and collaborating and focusing on priorities to provide a guarantee for urgent fundamental problems. On the other hand, western provinces' local fiscal construction funds are limited due to their inability to cope effectively with their finances. This requires the transfer of central government support, strengthening of economically underdeveloped regions in terms of social disability risk to cope with the financial support, and efforts to promote the equalization of public service, gradually narrowing the gap between measures and the strength of a region, in order to promote an overall reduction in social disability risk. Again, concerning regions with different degrees of social disability risk and risk performance dimension scores, enthusiasm, and initiative, regions must be encouraged, according to their respective social disability risks, to rely on their resource endowment, utilizing their advantages and taking targeted measures to establish their ways of dealing with social disability risk.

This study has several strengths. Firstly, we break through the existing research on the plight of disabled, older people and the lack of social support, and explore the construction of an evaluation system to assess and measure the degree of risk of disability among the older population in a country or region, bridging the research gap on the degree of risk of disability in regional societies and beginning to explore this area of research. Secondly, when assigning the indicators, we adopt a combination of subjective and objective assignments to eliminate the disadvantages of using only one assigning method. Third, after the evaluation system and indicator weights were determined, we also conducted an empirical study using authoritative data to analyze the degree of social disablement risk in China and make recommendations.

There are still some things that could be improved in this study. First, the regional degree of social disability caused by the significant risk of disability and semi-disability in the older population has not been studied by Chinese and international academics. This study is exploratory, and due to the limitation of team capacity, no clear definition and concept of disabled society and risk of social disability have been given. In the future, we will continue the theoretical research in this area and carry out further theoretical and conceptual clarification. Second, due to data availability, we only studied the regional degree of social disability risk caused by the extension of life expectancy and physical function decline in China's older population aged 65 years and above. However, severe disabilities and intellectual disabilities of newborns will also lead to an increase in regional social disability risk. In the future, we will continue to expand the research indicators related to the regional risk of social disability for further exploration and interpretation.

## 5. Conclusions

To sum up, population aging, as a long-term development process (70), is characterized by gradualness, hierarchy, and unevenness in the risk of economic and social development. Based on these characteristics and the objective fact that the increase in the number of disabled and semi-disabled older people in an aging society has different degrees of impact on different regions, this study constructs a regional social disability risk measurement index system. It also combines empirical data to measure the degree of social disability risk in 28 provinces in China. It was found that the risk of social disability in China is generally at a moderately high level and that the risk of disabling disability varies considerably between and within regions and provinces. As an exploratory study, this study has great significance and value as an exploratory study due to the lack of previous academic research on the degree of risk of such social disability.

## Data availability statement

The original contributions presented in the study are included in the article/supplementary material, further inquiries can be directed to the corresponding author.

## Author contributions

Conceptualization, methodology, and writing—review and editing: QG and YS. Software, formal analysis, resources, data curation, writing—original draft preparation, and visualization: QG. Validation and investigation: MF and ZL. Supervision, project administration, and funding acquisition: YS. All authors have read and agreed to the published version of the manuscript.
